# Real-Time Communication Support for Underwater Acoustic Sensor Networks †

**DOI:** 10.3390/s17071629

**Published:** 2017-07-14

**Authors:** Rodrigo Santos, Javier Orozco, Matias Micheletto, Sergio F. Ochoa, Roc Meseguer, Pere Millan, Carlos Molina

**Affiliations:** 1 Department of Electrical Engineering and Computers, Universidad Nacional del Sur, CONICET, Bahía Blanca 8000, Argentina; jadorozco@gmail.com (J.O.); matias.micheletto@uns.edu.ar (M.M.); 2 Computer Science Department, Universidad de Chile, Santiago 8370456, Chile; sochoa@dcc.uchile.cl; 3 Department of Computer Architecture, Universitat Politècnica de Catalunya, Barcelona 08034, Spain; meseguer@ac.upc.edu; 4 Department of Computer Engineering and Mathematics, Universitat Rovira i Virgili, Tarragona 43007, Spain; pere.millan@urv.cat (P.M.); carlos.molina@urv.cat (C.M.)

**Keywords:** underwater sensor networks, underwater monitoring, MAC protocol, acoustic transmission, submarine ubiquitous applications

## Abstract

Underwater sensor networks represent an important and promising field of research due to the large diversity of underwater ubiquitous applications that can be supported by these networks, e.g., systems that deliver tsunami and oil spill warnings, or monitor submarine ecosystems. Most of these monitoring and warning systems require real-time communication in wide area networks that have a low density of nodes. The underwater communication medium involved in these networks is very harsh and imposes strong restrictions to the communication process. In this scenario, the real-time transmission of information is done mainly using acoustic signals, since the network nodes are not physically close. The features of the communication scenario and the requirements of the communication process represent major challenges for designers of both, communication protocols and monitoring and warning systems. The lack of models to represent these networks is the main stumbling block for the proliferation of underwater ubiquitous systems. This paper presents a real-time communication model for underwater acoustic sensor networks (UW-ASN) that are designed to cover wide areas with a low density of nodes, using any-to-any communication. This model is analytic, considers two solution approaches for scheduling the real-time messages, and provides a time-constraint analysis for the network performance. Using this model, the designers of protocols and underwater ubiquitous systems can quickly prototype and evaluate their solutions in an evolving way, in order to determine the best solution to the problem being addressed. The suitability of the proposal is illustrated with a case study that shows the performance of a UW-ASN under several initial conditions. This is the first analytic model for representing real-time communication in this type of network, and therefore, it opens the door for the development of underwater ubiquitous systems for several application scenarios.

## 1. Introduction

The ocean is a source of life and energy that covers over two thirds of planet Earth, and about 80% of the whole live species are there. The life of the planet depends on resources that come directly or indirectly from the ocean, therefore it is important to take care of them. Many of these resources are fragile, e.g., UNESCO reports that half of the species living in the sea will be in danger of extinction within the next century [1].

These needs and opportunities in recent years have boosted research in underwater wireless sensor networks (UWSN), as a way to increase the feasibility of implementing systems that help us not only take care of the ocean and its resources, but also to identify hazardous events originating in the ocean that endanger the people living on the seashore [2]. The research in UWSN has been highly challenging due to the harshness of the submarine envirenment (i.e., the hostility of such a communication scenario) and the difficulties in transmitting information in the water [3]. These challenges refer mainly to the three first layers of the ISO/OSI model [4].

The communication scenario considered in this paper is oriented to cover a wide area in which several sensors are deployed. Once the sensors are anchored, the network topology is discovered and the clocks are synchronized. The nodes are in charge of sensing different parameters or capturing events. However, the data volume involved in the transmission is low. Messages are transferred in packets of at most 256 bytes including the header, and one packet is enough to send/receive all the information collected by the sensor. Another important issue is that the transmission speed is not high. Data rates are inversely proportional to the distance to cover, and as the network is deployed in medium to large areas, the transmission rate is set to 9600 bits/s according to recommendations of previous works [5,6,7]. Usual data transfers ratios are set between 5000 and 20,000 bits/s depending on the distance between nodes and available power. This communication scenario is typical in environmental monitoring or tsunami warning systems.

The research results in radio-frequency electromagnetic signals to support underwater communication were not entirely successful, particularly in sea water, where the conductivity of the medium is high. Provided that these signals do not propagate well in such a medium, a huge amount of power is required to transmit messages even for short distances. On the other hand, the presence of moving particles and obstacles in the propagation medium hinders the use of optical carriers. Although this communication type can achieve high transfer rates in the order of GB per second with a blue laser diode, the distances between transmitter and receiver should be short [8], which is not suitable to support the application scenario addressed in this paper; i.e., wide area networks that have low density of nodes and use an any-to-any communication scheme.

Although acoustic transmissions have been studied for decades [9,10], just in the last decade it has been shown that they are best option to support underwater communication [11,12], which has given room to develop UnderWater Acoustic Sensor Networks (UW-ASN). However, underwater acoustic transmissions also have several problems, e.g., the signal is attenuated while propagating, there are reflections in the bottom and surface, and currents and objects may introduce Doppler effects. The medium is so difficult to address that messages should be short, as long ones are more error-prone. Considering these characteristics, it is necessary to incorporate some kind of redundancy and fault tolerance to the communication process.

Most ubiquitous systems used to perform submarine monitoring or detection of hazardous events in the sea (e.g., tsunamis or oil spills) need to count on real-time communication. This communication type requires not only that messages are transmitted properly, but also that they reach their destination before a particular deadline. If the deadline is missed, the message is no longer valid and may have serious consequences for the communication process [13].

A feasible real-time schedule is one in which all messages comply with their deadlines. Real-time message scheduling in multi-hop networks (like UW-ASN) is a complex problem that requires the use of routing and queuing techniques. If all nodes in the network have a direct link to the rest of the nodes, the problem may be solved using an integer linear programming (ILP) approach. However, when a message should go through intermediate nodes, we have to decide not only when a node should transmit (i.e., a medium access control problem), but also how to select the appropriate path. In this case, the shortest path is not always the best one, as a per node scheduling should be incorporated in the analysis. In fact, a node holding more than one message has to schedule their transmissions, thus introducing additional delays.

In a recent study, we proposed a network model and a simple distributed medium access control (MAC) protocol for the case of UW-ASN [14] that helps address part of this problem. The network is modeled as a tree with a sonobuoy as root, and sensors as leaves. The information flows from the leaves to the root using intermediate nodes for aggregating the information collected from the previous layer of the tree. The synchronization process is performed in a hierarchical way, from the root to the leaves. At each layer, a synchronizing node is selected following certain rules, and these nodes are in charge of aggregating messages during the data transmission stage. The proposed algorithm considered the possibility of re-configuring the tree periodically. However, this working strategy was not designed to operate under real-time constraints, and the possibility of transferring data between any pair of nodes was not evaluated.

In this paper, we extend such a network model and also the algorithm proposed in [14], in order to include real-time constraints and message transfers between any pair of nodes in the system. The article also proposes a Time Division Multiple Access (TDMA) protocol based on a genetic algorithm, which includes an off-line allocation and scheduling algorithm. The feasibility conditions are given for the system to operate with hard real-time constraints. The reflected signals are used as backup, and an epidemic routing is evaluated as a strategy to improve the probability of conducting a correct message propagation, by introducing thus an opportunistic behavior in the network to provide fault tolerance.

The suitability of the proposal is evaluated using simulations. For that purpose, a framework was developed using SimPy [15]. In the simulations we measured some important aspects like the throughput and goodput, messages end-to-end delay, fault tolerance, and real-time behavior. The results show that the chosen routing policy and the priority management of the queues in intermediate nodes provide a robust system, even in the case of faulty nodes or links. The network behavior is within the theoretical computed margins verifying the validity of the analytic approach. The simulation results show that the UW-ASN can be used by different applications providing enough reliability. This is an important result, as this kind of network may be used to support pervasive systems and also ambient intelligence scenarios.

On the other hand, it is important to remark that the model proposed for UW-ASN is analytic, therefore the network performance evaluation can be automated allowing designers of both, protocols and underwater ubiquitous systems, to model their solutions (and evolve them) with minimal effort. Thus, these people can follow a prototyping-evaluation-tuning cycle until the solution is suitable enough to be implemented. This dynamic for modeling UW-ASN should help reduce development costs and risks, and increase the effectiveness of the resulting systems and protocols.

Next section discusses the related work. Section 3 describes the main design decisions made for implementing UW-ASN, and we justify the decisions made about the medium access control, message routing, and fault-tolerance protocols. Section 4 presents the system model and describes its components; e.g., the physical propagation model, the slot allocation model and the real-time model. Section 5 discusses the scheduling aspects by means of an example. Section 6 presents a meta-heuristic approach to solving the NP-hard problem of the slot allocation. Moreover, it shows several network topologies that are used to compare the performance of the proposed slot allocation algorithm, versus an integer linear programming solution. Section 7 describes a case study based on several simulations, which shows the network performance under various initial conditions. Such a section introduces the simulation tool, the obtained results, and it also compares the network behavior using the proposed model, with the shortest-path-first routing approach. Finally, Section 8 presents the conclusions and the future work.

## 2. Related Work

Underwater acoustic sensor networks have been proposed as a convenient way of supporting ubiquitous applications, ranging from biological, geophysical and environmental monitoring, to intrusion detection and also military uses that demand underwater communication. Radio, microwave, optical, and acoustic communication channels have been developed, though each technology has its own advantages and limitations. for instance, radio and microwave communications can provide high data transfer rates, but due to the seawater conductivity, they suffer from strong attenuation that increases with the carrier frequency, and demands a transmission power in the order of tens of kilowatts to cover tens of kilometers [8]. The German “Goliath” transmitter, developed during World War II, was capable of outputting up to 2 Mega-Watts of power to reach submarines in the Indian Ocean [16]. However, with new antenna designs, radio waves within the frequency range of 1–20 MHz can propagate over distances up to 100 m (at rates beyond 1 Mbps), using dipole radiation with transmission powers in the order of 100 Watts. Optical waves provide data rates higher than radio waves, but they are subject to attenuation due to the turbidity and dispersion of light produced by particles suspended in the sea water. Recent developments in blue laser diodes enable bit rates up to 7.2 GHz, but in very short distances limited to 6.8 m [8]. In counterpart, acoustic waves reach long distances in the order of a few kilometers, with a moderate power consumption at the expense of a low data bandwidth.

There are numerous research works that address different aspects of a UW-ASN, and here we mention those that in our opinion are the most relevant. It is important to note that in most cases, these research works are theoretical proposals that have been validated by analytical methods, simulations, and small scale experiments. The literature also reports experiments in real-world scenarios, which usually involve message exchange between two or three nodes. These experiments are oriented to provide solutions at the physical layer (e.g., they are for acoustic modem design or channel evaluation) and not at the higher layers (e.g., the link, network or transport layers).

The UW-ASN have several aspects to be considered that have not yet been universally defined or standardized, such as, the physical layer transmission mode, the protocols in the link, network and transport layers [6,7,17,18,19]. The literature shows four main approaches for medium access control (MAC): Frequency Division Multiple Access (FDMA), Carrier Sense Multiple Access (CSMA), Code Division Multiple Access (CDMA) and Time Division Multiple Access (TDMA). FDMA divides the carrier frequency into sub-bands assigning each one to an individual user among the neighboring nodes. Due to the limited bandwidth of underwater acoustic channels and its vulnerability to a multipath, FDMA is considered not entirely suitable [20] for sea underwater networks, especially in shallow water. However, some solutions for special topologies and node deployment restrictions use FDMA for UW-ASNs [21,22]. CSMA is a contention protocol in which the nodes should sense the presence of the carrier in the channel before accessing it. As underwater acoustic signal propagation delay is long, the carrier sense cannot indicate the real status of the channel, thus a node may start its transmission and detect it after the collision. To avoid this, nodes lock the channel with previous request to send (RTS) and clear to send (CTS) messages. Protocols derived from this are known as Multiple Access Collision Avoidance (MACA). This however reduces the throughput [23,24,25].

CDMA based MAC schemes have promising results and researchers focus on controlling the transmission power at all sensor nodes, in order to deal with the near-far problem and save energy. In [26] a hierarchical tree-topology is considered, where the nodes in same hierarchical level are multiplexed by means of different orthogonal codes. In order to preserve the nodes’ energy, the authors propose a CDMA protocol based on a repeated cycle of sleep/wake periods, reducing the power consumption of idle listening. The network topological restrictions were relaxed in [27], proposing a solution suitable for different network sizes and architectures.

A protocol named cascading multi-hop reservation and transmission (CMRT) proposes that intermediate nodes between a source and a destination may start handshaking in advance for the next-hop relaying before handshaking for the previous node is completed [23]. By this concurrent relaying, control packet exchange and data delivery cascade down to the destination. In [28] the authors compare analytically the slotted protocol FAMA with four way handshaking and the contention resolution Tone-Lohi protocol for different performance parameters, like energy consumption and throughput.

In case of TDMA, it divides a time interval (called a frame) into time slots instead of dividing the frequency band. Each slot is assigned to an individual node or a set of nodes whose transmissions do not interfere with each other. Collisions of packets from adjacent time slots are prevented by adding guard times. Large propagation delays, delay variance over the acoustic channels and the nodes clocks drift, all require the addition of guard time periods to minimize the probability of collisions in data transmissions. There are many TDMA-based MAC protocols proposed in the literature to overcome the shortcomings of the TDMA, which is considered one of the best multiple access techniques for UW-ASNs, due to its simplicity and flexibility [20].

In order to cover large areas, underwater applications require a multi-hop network. Many routing protocols have been proposed for UW-ASNs, which may include suitable media access control, network and transport protocols. These protocols implement two classes of routing: localization-based and localization-free. The first one can take advantage of the absolute and relative localization of nodes; however, the nodes’ position are not ease to know and maintain, since some nodes are fixed in depth and other are mobile, and unfortunately, GPS services are unavailable for underwater nodes. The second type of routing protocols is highly considered by the research community, and most of the proposals are oriented to communicate underwater nodes to a surface node (e.g., a sonobuoy), which is in charge of forwarding packets to an onshore station via RF transmissions [14,29,30,31,32,33].

In [34] the authors propose a routing protocol called DBR (Depth-Based Routing) that uses the depth sensed by each node, as a routing metric. DBR suffers from redundant packet transmissions, therefore it is energy consuming. An alternative dynamic routing protocol is presented in [35], based on a node address called HopID, which is related to the hop count from the source to destination node. In both protocols, some nodes could be used to relay messages more frequently than others, making their batteries get exhausted and creating thus blank zones and routing holes.

In critical applications based on UW-ASNs, like those used to deliver early warnings in hazardous events (e.g., tsunamis), a maximum end-to-end communication delay must be guaranteed. This requirement could be more critical than the energy consumption, therefore a real-time protocol must be used. Many proposals are trying to deal with this problem from different perspectives, for instance trying to minimize the end-to-end latency, increase the throughput, increase the link reliability, optimize synchronization, and increase the network efficiency. Although all these results are useful to improve the network performance, none of them ensure an upper-bounded message delay [36,37,38,39,40,41].

In [42] the authors addressed the problem of data transmission with an associated end-to-end packet deadline. Even if the proposal seems to be similar at first sight, they do not propose a MAC protocol, and the routing they use is based on probabilities; these are very important differences. The MAC introduces a delay that depends on the kind of protocol that is used. In that paper a contention mechanism is used, which has unbounded delays in the worst case. For choosing the next hop in intermediary nodes, the authors proposed a probabilistic approach, routing the message through the path with greater probability of reaching the destination node. In order to reduce the energy consumption, alternative paths are followed only when a fault is detected. In our proposal, an epidemic routing is used to provide the maximum possible degree of redundancy in a harsh medium very prone to interference and errors.

In [43] we present a real-time analysis for an underwater acoustic wireless network. The shortest path between a source and a sink node is used as routing policy, combined with a message or node slot allocation procedure in an optimized TDMA frame. Based on this, the schedulability condition is presented for the case in which messages are transmitted following a FIFO policy in the relay nodes.

In order to help address the real-time communication in UW-ASN, we propose a MAC protocol and routing model for underwater acoustic sensors networks, evaluating their performance through simulations. We recognize that the submarine medium is rather difficult to model and the actual implementation of the proposal would require adjustment to accurately represent the real message exchange in such a scenario. In order to illustrate such a challenge, in [44] the authors report the deployment of an underwater acoustic network in a real-world scenario, and in [45] they emphasize the impact that the modem characteristics have on practical underwater MAC design. These researchers also indicate the acoustic modems require a long synchronization preamble, and that synchronization time depends on the modem. Therefore, the model proposed in this paper should be tuned, mainly in the slot length definition, according to the hardware used in the network implementation.

## 3. Initial Design Decisions for Implementing UW-ASN

Applications based on UW-ASN are being deployed with different purposes as indicated in Section 1. These networks require to count on a gateway to conventional networks, which can be a buoy in the surface or a fiber link connected to some point in the coast. In most cases, the usual topology of a UW-ASN is based on a star or tree, where the root sensor (e.g., a sonobuoy) is the master and all the other nodes act as slaves. Although useful, this approach centralizes the communication and restricts the information flow, overloading certain nodes with much more traffic than others, since they have to aggregate the messages from downstream nodes [14]. This centralized model makes the system error-prone, since a failure in a root node or in the links to it, can produce a system failure.

In this sense, using a mesh network has several advantages because various nodes may act as gateways to traditional networks, and the information may flow through different paths. Besides, the possibility of sharing information between any pair of nodes contributes to the development of distributed applications or information processing, by increasing thus the computational power of the network and improving its performance. The integration and collaboration between nodes of a UW-ASN need to count on solutions to share data in a peer-to-peer scheme. Supporting this type of interaction allows the applications to implement distributed data storage, fault tolerance, rapid data sharing, and early warning propagation in the application layer [46].

Similar to the regular wireless sensors networks, the most common MAC protocols for UW-ASN can be grouped into two classes: contention (CSMA and MACA) and schedule (FDMA, CDMA, TDMA). In the first one, nodes transmit whenever they are able to lock the shared channel. For real-time messages this approach is not useful, since message delays may be unbounded. In the second approach, the FDMA is not useful as explained in the previous section, CDMA is promising and TDMA has been widely accepted. Although TDMA may introduce an important latency, the worst case delay may be computed and the behavior of the network (in terms of time) is predictable. TDMA can be divided into two modes. The first one considers that each node sends a broadcast to every node within transmission range. In this case, if a node has several messages to transmit to different destinations, it has to wait for equal number of frames. In the second mode, a per-message TDMA is computed in such a way that the slot for sending a message from node *a* to node *b* is defined, and also the moment at which node *b* receives the message from node *a*. In this case, each message has a particular slot to be sent and received at destination, and the nodes may wait for the proper instants to become active.

Using TDMA in UW-ASN has several advantages since, e.g., each node transmits and receives in specific time slots (or time windows), where the channel is free to transport messages without collisions with other nodes in the network, or it is able to receive messages from specific nodes. The sequence of slots is periodic, and each set is named frame. In a frame, all nodes in the network transmit and receive without interference from other nodes. The slot allocation is an NP-hard problem [47,48]; however, in this paper we propose two solutions to address such a problem: one based on ILP techniques (like branch-and-cut) and another based on evolutionary meta-heuristics (like genetic algorithms).

Provided that in a mesh network the routing is important, as it is the access to the transmission channel, we propose to use epidemic routing to address this challenge. The harshness of underwater communications requires to employ all possible strategies to reduce the probability of failure. Therefore, replicating the messages in several paths, increments the chances of achieving a successful transmission.

As mentioned before, real-time messages should be received before their deadlines; therefore, managing the delays is a key design aspect in these networks. These delays have two major sources: one is originated in the time needed by a message to go from the source to the destination node, and the second is represented by the delay a message may have in each hop, since several messages may be in queue and some scheduling policy should be implemented. In the first case, the delay time is determined by the number of hops, the sound speed in the water, and the distance between the nodes. These are physical parameters that are independent of the implemented transmission policies. For determining the second delay case, we propose to use a deadline monotonic scheduling policy for which we compute the worst case delay. Based on it, we provide a feasibility test for real-time messages.

## 4. System Model

Although underwater sound propagation has been studied in the literature and the physics are well-known, actually establishing a link between two nodes continue being quite difficult. Next we present the models proposed to represent the physical propagation of the messages, the network (including the nodes synchronization), the real-time message transmission, and the message routing.

### 4.1. Physical Model

Given the underwater sound propagation is subject to attenuation, the path loss depends on several factors. Some of them are related to the communication medium, e.g., water temperature, density, and pressure, while others depend on the signal being transmitted. In the last case, the most important parameter is the frequency of the signal that impacts directly on the absorption. As the sound propagates in the water, there is a spreading loss that increases with distance. The most accepted model for the path loss is given by [49,50,51], represented by the following equation:

(1) $$A\left(l,f\right)={\left(\frac{l}{l_{r}}\right)}^{k}a{\left(f\right)}^{l-l_{r}}$$

where *f* is the signal frequency, and *l* is the transmission distance taken in reference to some *r*. The path loss exponent *k* models the spreading loss, and its usual values are between 1 and 2 (for cylindrical and spherical spreading, respectively). The absorption coefficient a(f) is an increasing function of frequency, which can be obtained using an empirical formula [52].

**Figure 1:**
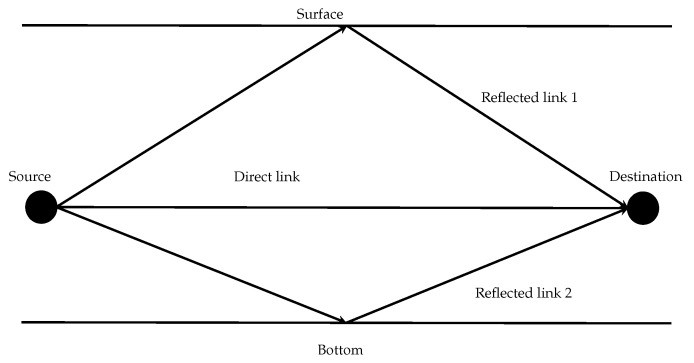
Example of multipath transmission.

Equation (1) assumes that the transmission path between the source and the sink is straight and free from interference. However, underwater acoustic signals propagate in a physical channel with boundaries in the surface and the bottom (Figure 1). Usually, part of the signal is reflected in both, generating replicas that arrive to the sink at different instants. This effect, known as multipath, makes that the surface reflects the signal without loss, but in the bottom the signal is attenuated. This attenuation depends on the kind of material present in the floor (e.g., sand, rocks or mud).

The reflected messages have to cover longer distances before reaching the sink (given by Equation (1)). Given the path loss is proportional to the distance, the reflected signal arrives more attenuated to the sink. Anyway, depending on the distance and the network deployment, multiple copies of a message may arrive successively to destination with enough strength to disturb other messages.

The initial versions of the UW-ASN model (particularly, those described in [14,43,53]) ignored the multipath effect in order to focus on the medium access control protocol, and we have assumed a symmetrical propagation delay between any pair of nodes. In this paper, we extend the model to propose a directed multipath that accounts for the multipath problem, and incorporates the possibility of introducing different delays according to the direction in which a message is propagated. There are several causes for this but, to mention just one, the presence of currents contribute in one direction to the propagation speed, while increase the delay in the opposite.

Acoustic signals are longitudinal waves. In the case a collision among more than one signal is produced, there is a transitory composition of the waves at the point of collision; but after this, the signals continue their propagation undisturbed. Clearly, collisions should be avoided at the sinks, due to they may corrupt the messages in a way that they cannot be recovered.

### 4.2. Network Model

At this point it is useful to consider time as a discrete variable. Time can be seen as a sequence of slots *t* numbered {1,2,…,n}. Even if time is continuous, in order to handle nodes accessing to the transmission channel, the division in time slots facilitates the allocation and provides an efficient tool to separate possible transmission and reception interference within the nodes. Under this approach, the slot duration is a design decision that considers silent intervals, both at the beginning and at the end of it. These intervals account for variations in the transmission propagation speed, for example, caused by currents or for the need to turn off the receiver while transmitting. In this way, a node transmits a message during the slot, but it does not mean that all the time is used for the transmission. This concept is explained for the case of frame boundaries as guard times in [36]. In previous UW-ASN TDMA proposals, the slot time included the longest propagation delay. Although this guarantees that no collisions are produced, for the case of acoustic underwater transmissions where propagation time is usually higher than packet length, this approach wastes too much time [48,54,55].

Considering the previous definitions, the network can be modeled as a directed graph G=(V,E), in which *V* is the set of network nodes and *E* is the set of edges (i.e., links between network nodes). If two nodes *u* and *v* are within transmission range, there are two sets of edges connecting them $\Theta \left(u,v\right)=\{e_{h}\left(u,v\right)\}$ and $\Theta \left(v,u\right)=\{e_{h}\left(v,u\right)\}$, where *h* numerates the links. Each edge of the graph has a label that represents the transmission delay between the nodes measured in *time slots* ($\tau_{uvh}$). The set of nodes that have a direct link with i∈V is the neighbor set, and it is denoted N(i). The multipath effect described in previous section is modeled with many edges with different weights among two neighbor nodes representing the different paths and propagation delays. Table 1 shows the notation used in the model.

As collisions are important only if they are produced at the node, there are four different scenarios to consider as stated in [36]. The first scenario is when two messages arrive simultaneously to a node; this case is named the Rx-Rx. The second scenario is produced when two messages tried to be transmitted simultaneously in a node; this is the Tx-Tx case. The third scenario is when a message is transmitted at the time that another message is being received; this is the Tx-Rx case. The last one is named the Rx-Interference case and it arises when a message interferes another one in a node. The interfering message has a different destination node; this last case is similar to the Rx-Rx.

In the case of the multipath, the collision can be produced with any of the message replicas, therefore special care in the slot time assignment should be taken. As the transmission medium is water and the signal is acoustic, the presence of several edges between two neighbor nodes does not imply independent paths; all of them are used whenever a node transmits.

In [43] we presented an integer linear programming (ILP) model to minimize the frame length. However, such a model does not consider the transmission delay, and different solutions with the same objective function value have different frame sizes. In [48] the authors proposed a TDMA scheme similar to the one proposed in this paper, but they use continuous time. They named the graph model as the local conflict graph problem and show that it is a NP-Hard problem; however, they do not consider the multipath effect. In this paper, we modify this previous model to include the maximum delay in the objective function, and with this, the minimum frame (if exists) is found. If we note $m_{i}\in N$ the slot in which node *i* transmits, and *L* the frame length, we can minimize *L* as follows:

(2) MinimizeL

Subject to:

(3) $$\begin{array}{cccc} L-t(i) & \geq \max\tau_{ijh} & & \forall j\in N(i);\forall h\in \Theta (i,j) \end{array}$$

(4) $$\begin{array}{cccc} t_{i}-t_{j}-M\delta_{ij} & \geq \tau_{ijh}+1-M & & \forall i\in V;\forall j\in N(i);\forall h\in \Theta (i,j) \end{array}$$

(5) $$\begin{array}{cccc} t_{i}-t_{j}-M\delta_{ij} & \leq \tau_{ijh}-1 & & \forall i\in V;\forall j\in N(i);\forall h\in \Theta (i,j) \end{array}$$

(6) $$\begin{array}{cccc} t_{j}-t_{k}-M\omega_{ijk} & \geq \tau_{ikh}-\tau_{ijg}+1-M & & \forall i\in V;\forall j,k\in N(i);\forall h\in \Theta (i,k);\forall g\in \Theta (i,j) \end{array}$$

(7) $$\begin{array}{cccc} t_{j}-t_{k}-M\omega_{ijk} & \leq \tau_{ikh}-\tau_{ijg}-1 & & \forall i\in V;\forall j,k\in N(i);\forall h\in \Theta (i,k);\forall g\in \Theta (i,j) \end{array}$$

(8) $$\begin{array}{cccc} \delta_{ij} & \in \{0,1\} & & \forall i\in V;\forall j\in N(i) \end{array}$$

(9) $$\begin{array}{cccc} \omega_{ijk} & \in \{0,1\} & & \forall i\in V;\forall j,k\in N(i) \end{array}$$

where *M* is a sufficiently large constant, and $\delta_{ij}$ and $\omega_{ijk}$ are binary variables that are used to implement the inequality conditions in the ILP solver, e.g., constraints (8) and (9). Constraint (3) limits the minimum length of the frame to the maximum transmission delay of the edges, in such a way that it considers both transmission and reception of the message in each neighbor node. Constraints (4) and (5) prevent the Tx-Rx conflict, making that messages arriving from neighbor nodes do not have to collide with the message being transmitted in the node. Constraints (6) and (7) are used to prevent collisions of type Rx-Rx. Thus, neighbor nodes should transmit their messages in such a way that they do not collide on any node within transmission range. In the case of these four constraints, all possible edges are explored to consider all possible multipath cases.

The use of TDMA supposes a strict clock synchronization and also the network topology must be known by the nodes. As mentioned in Section 1, in [14] the authors introduced a hierarchical synchronization protocol, where the network topology is discovered and the nodes learn about it. Once the node’s clocks have been synchronized and the slot assignment computed, message transmissions are used to keep updated the network topology. Within each message, clock synchronization messages can be included in the header together with information about the aliveness of the nodes.

### 4.3. Real-Time Message Model

In this paper, we assume that any node may transmit a message to any other node in the network, if there is a valid path between them. We denote as $m_{ij}$ a message from node *i* to node *j*. We also assume all messages require one time slot to be transmitted, and that they have associated a period $P_{ij}$. It may occur that the activity in certain applications is sporadic. In that case, the period is associated to the minimum interval of time between two successive events. For the real-time analysis, if the node has no message to send, a short *hello* message is transmitted to keep both the network topology and synchronization updated. These messages are received by neighbors, but they are not propagated along the network.

A real-time message should be received before a certain instant, named deadline. The message is said to be hard real-time if missing a deadline has catastrophic consequences. For instance, in a tsunami warning system, the message alerting the arrival of waves should be received in the coast with enough time to allow the evacuation of the people in the potentially affected area. If some deadlines are allowed to be missed, the system is said to be soft real-time [56]. These cases are related to applications in which there are timing requirements, but these are not stringent. Nodes should implement some priority policy for scheduling the messages. In this paper, the Deadline Monotonic Scheduling is proposed. As there may be many different deadlines, messages are grouped for reducing the number of priority levels that nodes should handle [57]. In the proposed model considerations are taken to have some degree of redundancy to provide fault-tolerance in the case of transmissions failures [58].

In systems like those considered in this paper, deadlines $D_{ij}$ are usually larger than the periods, as messages take a long time before reaching the sink. With this, we define the set of messages as $Z=\{m_{ij}\left(P_{ij},D_{ij}\right)\}$. The deadline is used to stop propagating messages once it is clear they are not going to be received on time. This is implemented with a deadline stamp that is reduced in each hop. When the time needed to reach the destination is greater than the remaining time denoted by the deadline stamp, the message is discarded. This may happen in the case a node is overloaded with higher priority messages.

**Figure 2:**
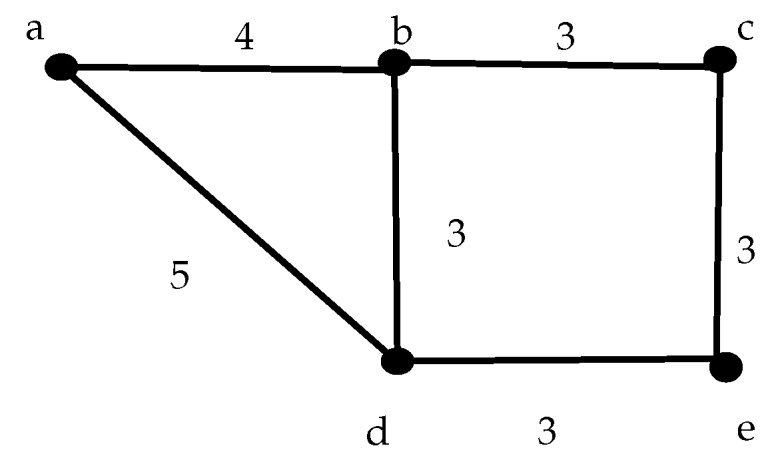
5-node network example.

As nodes can only transmit in the slot allocated in the frame, if there are more than one message in the queue, then they should use as many frames as necessary to send all the messages. In case that all nodes in a route have their queues empty, in the worst case, the end-to-end delay for a message will be the amount of necessary hops, plus one time the frame length. This is a necessary condition for the system to be schedulable. In case of the network presented in Figure 2 that has five nodes and six edges with the same delay in both directions (for simplicity), the minimum frame is the one presented in Table 2. As it can be seen, it has a length of six slots. For a message starting in node *a* and destination in node *e* (i.e., $m_{ae}$) there are two hops, therefore, at least three frames or 18 slots are needed.

Within each node, when there are more than one message to be sent, a scheduling policy should be followed to guarantee the deadlines.

### 4.4. Routing Model

In previous works, we have proposed a hierarchical propagation model [14] in which messages propagate in a tree from the root to the leaves, and later from the leaves to the root with nodes aggregating the messages from the sons before forwarding them to the next layer or father. In [43] the network was restructured as a mesh, and a routing algorithm based on the shortest path first (SPF) was proposed. Although valuable, these approaches do not consider the multipath problem and no fault-tolerance backup policy was proposed. The routing model we introduce in this paper takes advantage of the multipath effect, in order to provide a probabilistic recovery from transmission errors originated in unexpected disturbances. In this proposal we also have added an opportunistic routing based on the epidemic model.

In [43] the SPF was used to determine the best path for a message; however, underwater transmission is so vulnerable to disturbances that using just one route jeopardizes the possibilities of success. Therefore a *multicast* propagation is best suited in this case, since every message is forwarded from source to destination through different paths until it has been received. Deadlines associated to messages are used to discard those routes that are not feasible from the beginning. UW-ASN have a low traffic intensity and this allows the use of epidemic routing; otherwise, some nodes may be overloaded as they are in the middle of several paths.

In the slot time allocation, the replicas coming from multipath propagation are usually discarded, but their presence should be considered in the schedule to avoid collisions. Therefore, a message that has been corrupted in the direct path can be recovered from a replica arriving later. Although different approaches are feasible to do this, for the sake of simplicity we have assumed that the message is read again.

In the example represented in Figure 2, let us suppose that node *a* has to send a message to node *e*, and there are several available paths. The SPF is *a*—*d*—*e*, which has a worst case transmission delay of 18. However, if the link *a*—*d* is corrupted, the message is lost and the transmission fails. By using both *a*—*d* or *a*—*b*, the system is more robust and even if *a*—*d* fails, the message may survive in the link *a*—*b*. For this to be effective, neighbor nodes should overhear the activity in the network so they can detect a fallen link or a dead node. In the case of underwater acoustic channel, the probability of faults is significant. Therefore, to prevent the message lost, every node receiving a message will forward it. Although more energy is required to implement this kind of propagation, it is more robust. For instance, *a* sends a message to *e* through *d* with an epidemic policy. Node *b*, listens to *a* transmission and forwards the message to *e* through *c*. This message will also be received by *a* and *d*, but as they already have the message, they will not forward the copy. The node *e* will receive a maximum of two copies of the same message. In the case that the path *a*—*b*—*c*—*e* requires more time than the deadline, the whole path is discarded saving energy and allowing other messages to use that path.

In challenged networks, like UW-ASN, there is a high probability of failure in the transmissions. In WSN several protocols were proposed to provide alternative paths in case of nodes failures by overhearing transmissions [59]. However, in the underwater scenario it is very difficult to determine whether a path has been corrupted or the node is dead. In the previous example, node *b* knows that node *d* has received a message from *a*, and therefore, it has to forward the message. Node *b* knows this by *overhearing* the transmission, therefore, it now waits for node *d* to forward the message. If this does not happen, it has no way to know if node *d* is dead or the paths a−d or d−b have been blocked. It may also happen that node *d* has a long queue of messages waiting to be sent, by delaying the one from *a*. In this context, epidemic routing provides the highest probability of transmission. In [53] the authors analyze the performance of some opportunistic routing protocols considering average time to arrival and energy consumption in the network. In that paper it is shown that the energy consumption of the epidemic routing is not always worse than other approaches, as the time needed to deliver a message is shorter. The other aspect that should be considered is the amount of data to be transmitted. This kind of network has a low channel utilization. It is not used for transferring large amounts of data or provide Internet connectivity.

## 5. Scheduling and Real-Time Feasibility Conditions

UW-ASN share a common physical channel. In Section 4.2 we have discussed the computation of a minimum frame to guarantee that each node transmits and receives messages at specific slots. In the rest of the frame, the node may sleep thus saving energy. This is possible only after the network topology has been learned by the nodes, since they have conducted the synchronization process [14]. Real-time operation requires that messages arrive without errors and within their deadlines; in this section we present the necessary conditions for this.

On the other hand, in Section 4.4 we described the epidemic routing to provide redundancy in the transmission. In each frame, the message advances one hop from the source to destination following different paths. At this point is where the scheduling policy in the nodes becomes important. A node may receive during one frame a message from every neighbor and besides it may generate its own messages. A message may be delayed within node *i* for $\delta_{i}$ frames depending on the load. A policy to order the message transmission is necessary in order to bound delta.

In the worst case, the end-to-end delay for a message from node *a* to node *b* following path *j* with *n* hops is given by the following expression:

(10) $$\Delta_{ab}^{j}=T_{f}\left(\left(n+1\right)+\sum_{i=1}^{n+1}\delta_{i}\right)$$

Equation (10) has two terms. The first one computes the delay associated to the path used. The most common solutions for path discovery are based on Dijkstra algorithm to determine the shortest path from any node in the network to any other node (SPF, shortest path first), or the Bellman-Ford distance vector algorithm. In the present case, the computation of the shortest path is more simple as in each frame duration ($T_{f}$) the message completes one hop. If *n* is the number of hops in path *j*, a message being transmitted as soon as it is received requires n+1 frames to reach the destination node if it is generated just after the slot in which the source node has the chance to transmit. The shortest path is the one with less hops. Those paths that are not able to forward the message before its deadline are not followed and they can be used to forward other messages.

The second term in (10) refers to the waiting time in the queues of intermediary nodes. Average waiting time in the queue may be computed from Little’s theorem [60], but to bound it, it is necessary to compute the response time according to the scheduling policy used in the node. The best path for underwater networks is not necessary the one with lower delay in the transmission, as nodes may be overloaded with messages and long delays may take place in each hop. Energy demand is another aspect that should be considered as underwater nodes depend on batteries and these are not easily replaceable. This is a strong reason to use alternative paths even if the transmission has long delays.

The most simple scheduling policy in the nodes is First-In, First-Out (FIFO), but it may happen that a message with a higher priority is postponed in favor of another one with a lower priority. Real-time scheduling policies have been studied in the past and different feasibility conditions and response time analysis have been proved for them. In this paper, we assume a periodic monotonic scheduling policy (RMS) in which higher priorities are given to tasks with shortest periods.

In [61] the response time for a set of *m* periodic, independent and simultaneously released messages is proved to be given by:

(11) $$\min R\;\;\;s.t.\;\;\;R=\sum_{h=1}^{m}\left\lceil \frac{R}{T_{h}}\right\rceil$$

where R∈{1,2,…}.

Equation (11) is solved iteratively. A solution exists if and only if:

(12) $$U=\sum_{h=1}^{m}\frac{1}{T_{h}}\leq 1$$

Condition (12) considers that the message can be transmitted at any instant. In the case of the UW-ASN proposed in this paper, the possibility of accessing the channel is limited to the allocated slot within the frame. Therefore, the condition and response time should be reformulated to count the reduction in the bandwidth and the frame granularity imposed by the TDMA:

(13) $$\begin{array}{c} U=\sum_{h=1}^{m}\frac{1}{T_{h}}\leq 1/T_{f} \end{array}$$

(14) $$\begin{array}{c} \min\delta \;\;\;s.t.\;\;\;\delta =T_{f}\sum_{h=1}^{m}\left\lceil \frac{\delta }{T_{h}}\right\rceil \end{array}$$

**Figure 3:**
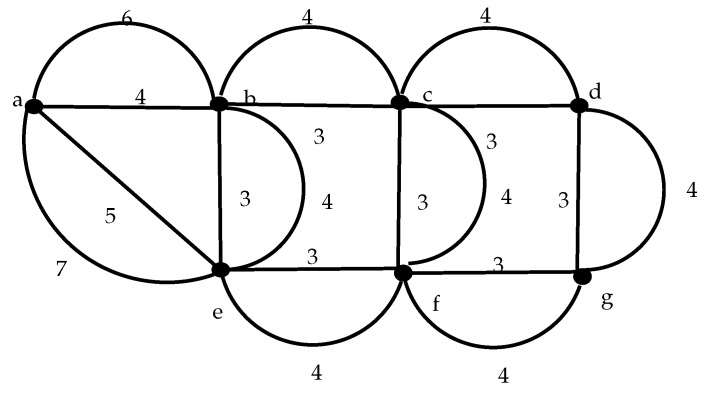
7-node network example with multipath.

Example:

In order to illustrate the different aspects discussed in the previous sections, next we present a simple example. Figure 3 shows a seven node network with the labels in the edges denoting the transmission delay between adjacent nodes. We added in this case an additional path between each pair of nodes that represents a delayed arrival, multipath transmission. For simplicity of presentation, we assume that the delay in both directions is the same. The solution to the ILP model produces a frame of 11 slots in which all nodes transmit and receive. Table 3 shows the slot allocation.

Let us suppose that node *a* has to send a message to node *g*. According to the proposed epidemic routing, it implies that the message is received by nodes *b* and *e* in slots 10 and 11 respectively. Once the message is received, both nodes will forward it to all their neighbors. In this case, both nodes *e* and *b* will forward the message in slot 12. At this point it is interesting to mention that the message will be received by node *a* in slots 17, 18, 19 and 20. The node will know in this way that its message has been forwarded by both nodes. Also nodes *b* and *e* will receive the copy of the message sent by each other, but they will discard it as it has been already received. Nodes *c* and *f* will receive the message at slot 17. These nodes have to wait for the next frame to forward the message, in this case until slot 25. The message is finally received at node *g* coming from node *f* at slot 29, while the other branch still requires another frame as the message is received by node *d* in slot 29, and in the next frame at slot 38 will forward it to node *g*, that will receive the message at slot 42. We have assume for this analysis that the message was ready to be sent when node *a* was able to transmit in the first place. However, the worst case condition may happen when the message is generated just after the slot for node *a* has passed. In that case, another 11 slots should be added to the total delay. The worst case transmission time is then 40 and 53, respectively for each path. If the deadline associated to this message $D_{ae}\geq 53$, then both paths are feasible; in other case, if $40\leq D_{ae}\leq 52$ only the shortest path is feasible and if $D_{ae}\leq 39$, no path is feasible.

The previous analysis considers that there is only one message to be transmitted. However, this is not the case for a real network in which messages may be originating at different rates in the nodes and require to be sent to destination. In this situation, a message may have to wait for several frames before gaining access to the channel for transmission, as described in Equation (11).

Deadlines for the messages depend on the application constraints. For instance, in the case of tsunami warnings the message reporting the possibility of the tsunami hitting a particular spot at the coast, should arrive well ahead in order to allow people evacuation. For the purpose of this example, we have assumed the following parameters for the system. Let us suppose the following set of messages defined as stated in Section 4.3.

(15) $$\begin{array}{c} Z=\{m_{ag}\left(100,300\right),m_{bd}\left(80,200\right),m_{ce}\left(80,200\right),m_{db}\left(80,220\right),m_{ec}\left(80,200\right),m_{ga}\left(100,300\right)\} \end{array}$$

In Table 4 the worst case scenario for each node is described. For each one, all the possible messages that go through them are listed. With this information, Equation (14) is used to determine the worst response time for each message in every node.

In Table 5 the worst case response time in each node according to Equation (14) is shown:

Based on Table 5 and the number of hops in each path, the worst case end-to-end delay may be computed and compared with the deadline of the message. With this information, those routes that not satisfy the messages deadlines are discarded. In Table 6 all the possible paths for message $m_{ag}$ are shown, and the worst case delay is computed.

As it can be seen, the last route is not feasible, since node *c* will not forward $m_{ag}$ when it is received from node *f*.

## 6. Computing the Minimum Frame with a Genetic Algorithm

TDMA slot allocation has been well studied by the research community, and several algorithms based on the vertex coloring in a graph have been proposed to address this problem. The bandwidth reservation problem in a TDMA-based scheme is equivalent to the satisfiability (SAT) problem, which is known to be NP-complete [47,48]. Based on this, the use of meta-heuristics like genetic algorithms, may provide a good solution in less time than the required with ILP solvers. In this section, we present a genetic algorithm to compute a frame that guarantees collision-free transmissions and receptions in all nodes.

Given the number of nodes *N* of an UA-WSN, let Nodes[] be an array of *N* elements stating the order of allocation of nodes to the frame. Let the following algorithm be the frame allocation method from a given specified array Nodes[],

**Conjecture** **1.** *The frame of minimum length can be found by Algorithm 1, given as input a suitable ordering of array* Nodes[].

**Algorithm 1:** Minimum length algorithm in pseudo-code 

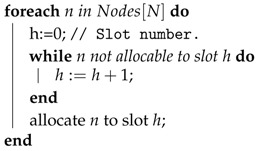



From assumption made in Algorithm 1, the optimum frame can be obtained by evaluating all possible permutations of array Nodes[]. Eventually, if the network is large, a high computational cost is required to evaluate all the permutations. As presented in Section 4, we can find the minimum frame with ILP techniques. Based on the proposed model, we developed an evolutionary optimization technique that follows the genetic algorithms approach, which is explained in the next subsections.

### 6.1. Genetic Algorithms

Genetic Algorithms (GA) were proposed to explain the adaptive processes of natural systems, as well as the design of artificial software systems that keep important mechanisms of the natural evolution. These algorithms consider a population of candidate solutions called chromosomes, and use an objective function to evaluate the life expectancy of these population members (i.e., the chromosomes). In our case, the objective function takes as an argument the vector that indicates the allocation order of nodes to the frame, computes the frame length according to the Algorithm 1, and returns the frame length as the quality of the solution found.

The GA operates through an iterative method, where in each iteration the population undergoes a series of transformations that lead to a new generation. The evolutionary process of each generation consists of a series of steps, also called probabilistic transition rules, which are briefly explained next.

#### 6.1.1. Selection and Parent Sampling

The selection scheme assigns to each individual of the population an expected number of descendants that is proportional to the probability of that individual to be selected. In the simple GA method, it is used propositional selection as sampling algorithm and stochastic sampling with replacement [62] or roulette wheel [63]. The selection probability assigned to each chromosome *i* is given by:

(16) $$\pi_{i}^{t}=\frac{f_{i}^{t}}{\sum_{i=1}^{p}f_{i}^{t}},i=1,...,m.$$

where *p* is the population number, *t* states the generation number, and $f_{i}^{t}$ indicates the quality of chromosome *i* at generation *t*. The cumulative probability of each chromosome is computed according to the following equation:

(17) $$\Pi_{i}^{t}=\sum_{j=1}^{i}\pi_{i}^{t},i=1,...,m.$$

Finally, the selection process is performed based on the $\Pi_{i}^{t}$ values of each chromosome, which represents a random choice in a roulette wheel, where each mark corresponds to each chromosome, but in which the separation between marks is proportional to the quality of each individual.

#### 6.1.2. Crossover and Mutation Operators

Since the optimization variable consists of an ordered array of elements, after the crossover operation between two solutions there must be no repeating elements in the offspring chromosome. To avoid this, different operators can be implemented, being partial-mapped crossover operator (PMX) the best known and the one used in this work [64]. Given a pair of selected parents for the crossover operation, a subsequence is established by defining two crossover points on both chromosomes, and by swapping this subsequences the resulting children contain the same exchanged subsequences of the parents. To eliminate repeated elements, a repair algorithm is used, so that the exchanged information is preserved, and all constraints of the problem are satisfied.

In this case, it can appear mutation consisting on adding random alterations to the chromosomes, so that new features emerge in the solutions, and the speed of convergence can be improved. For this particular problem, the mutation operator introduces modifications in chromosomes by swapping two randomly selected genes, and it takes place on different chromosomes according to a tuning parameter called mutation probability.

#### 6.1.3. Generational Substitution

After performing the selection-crossover-mutation process, the next step is to evaluate each chromosome and to determine which individuals will constitute the new population. Although there are different strategies to complete this step, in the simple GA that is used in this work, generational substitution of half of the worst-conditioned population is performed; that is, a temporary population equivalent to the sum of the current generation and the descendant are sorted according to the condition of each, and then half of the set is discarded. The chromosome population surviving the next generation results from competition between parents and descendants of the current generation.

#### 6.1.4. Termination

The termination condition of the algorithm can be defined by the user limiting the execution time or the number of generations, being the latter the most used since it is proportional to the number of evaluations of the objective function and it is a useful reference parameter for evaluating performance or making comparisons with other techniques. The result of the optimization method is the highest quality solution within the population of individuals of the last computed generation.

#### 6.1.5. Complexity

There are several items that contribute to the GA complexity. In what follows, the complexity (also known as cost) per generation or iteration of the GA is considered. The procedure has two distinct parts. In the first one, the fitness function has to be evaluated for each member of the population. The complexity of this part is given by the product of the population size (*p*) and the cube of the number of nodes in the network (*n*), i.e., $O(pn^{3})$. The selection process of GA includes sorting the members of the population according to the fitness value, but this process cost is O(plog(p)) in the worst case. The complexity per iteration is the combination of both terms, and depending on the relative values of *p* and *n*, this complexity is $O(p\left(\log\left(p\right)+n^{3}\right))$. The amount of iterations needed (*g*) may be important. The final complexity of the GA method proposed is given by $O(gp\left(\log\left(p\right)+n^{3}\right))$.

### 6.2. Effective Period

Although the frame length defines the system period, it is possible to reduce the transmission period of each node by overlapping each frame with the one from the next period.

**Definition** **1.** The effective period is defined as the minimum period between messages transmission from the same node that is possible by overlapping frames.

Overlapping is not always possible, because it depends on which slots are allocated at the beginning and at the end of the obtained frame. In some cases, a frame of greater length than the optimal frame may have a shorter effective period. In order to take this advantage into account, it is possible to modify the objective function of the problem that contains the Algorithm 1 in a way that instead of returning the length of the computed frame, it is returned the maximum possible overlapping slots, or what is the same, the effective period of the frame. In this way, the optimization heuristic can find frames of greater length, but of minimum effective period increasing the network throughput.

### 6.3. Results Obtained

**Figure 4:**
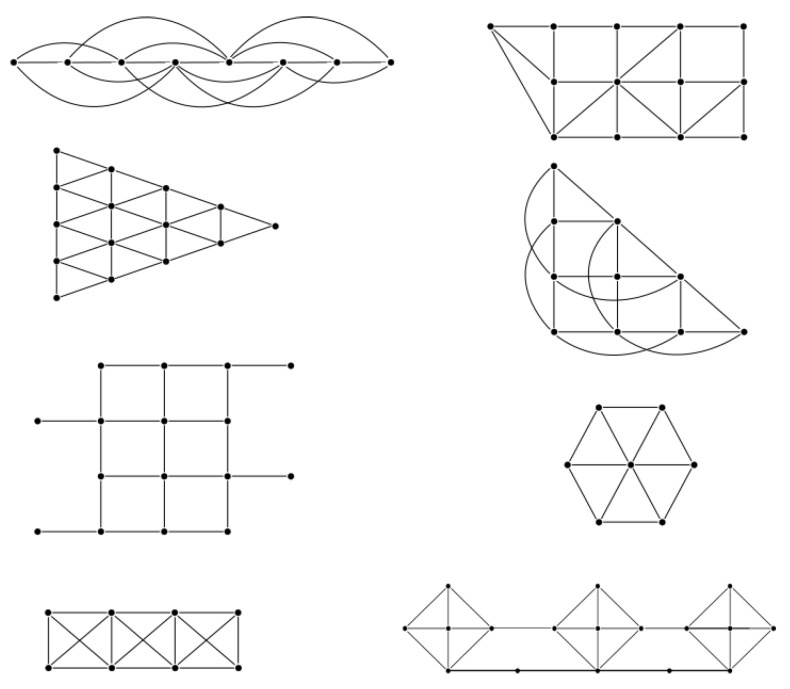
Different topologies evaluated in the simulations.

We have implemented the GA using standard C code running on a notebook with a i5 CPU, 4 GB RAM and Ubuntu 16.04 version as operating system. In all cases we use a 20 random arrays to produce the initial slot allocations, with PMX crossover technique and 0.3% probability of mutation. At the same time, we use the GLPK solver [65] to compute the minimum frame length using the ILP model. We choose small and medium size networks with different topology configurations. The layouts were thought for different applications since the distribution of nodes change significantly. In the majority of the cases, the GA achieved the same result as the ILP method, but consuming much less time. Table 7 shows the results for the configurations presented in Figure 4. What is more important is that using the GA can facilitate the overlapping mechanism to reduce the effective period of the network. All the computation times (CT) in the Table are given in milliseconds and the frame length in slots. The ILP method requires two and three orders of magnitude more time to find the optimal solution, and in the case of the second instance, the method was stopped after 3600 s without reaching a solution. The last two columns show the overlapped effective frame length computed with the improved genetic algorithm. The frame length, as we will show in Section 7, is important to the network performance.

## 7. Simulations

In this section we present several simulations that show the overall performance of the network under diverse initial conditions. We used the example of Section 5 to measure end-to-end delay, the load in the different nodes, and we introduced random faults to check the network reliability. In what follows we will explain with more details the experimental framework and the tests we performed to validate the model.

### 7.1. Experimental Framework

We developed a simulator based on SimPy [15], which is a process-based discrete-event simulation framework, implemented in the Python programming language. We modeled the nodes and underwater links. The basic features are low data-rate, large propagation-delay, multipath fading, and high error-probability. The medium access was implemented with TDMA with off-line slot-allocation within the frames.

Once the system is configured in the simulator, the experiments were run with diverse release-times for the messages in the source nodes. With these variations we can evaluate the network behavior, and the loads in the intermediary nodes for each path. Specifically, we measured the following four parameters:

1. End-to-End delay.
2. Packet Delivery Ratio (PDR), i.e., the ratio of packets successfully received, compared to the total sent.
3. Network Goodput Ratio, that is, the ratio of packets received before their deadline, compared to the total sent.
4. Number of messages held in the processing queue of nodes.

### 7.2. Validation of Simulations

For validation, we used the example presented in Section 5. In that example there are seven nodes and 18 edges, including the multipath reflections. The minimum frame-allocation computed using the ILP model is given in Table 3. As it can be seen, 11 slots are enough to fit all transmissions and receptions in the nodes. The set of messages considered is the one described in that section. However, the release time of the messages was changed, in order to evaluate the impact of messages arriving to intermediary nodes. The idea is to validate the real-time behavior of the network by producing overload conditions in those nodes, and thus delaying the transmission of messages.

**Figure 5:**
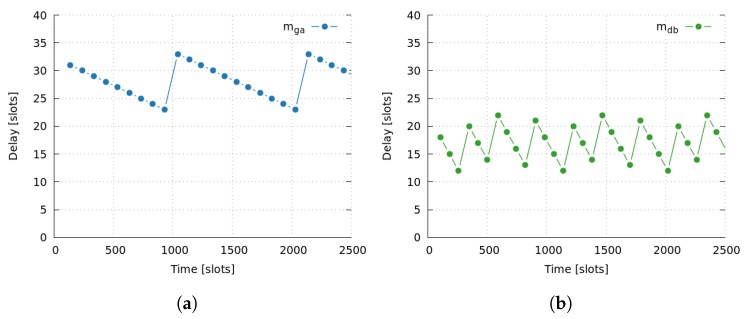
Example of end-to-end delays over time. (**a**) Messages from node *g* to node *a*; (**b**) Messages from node *d* to node *b*.

We simulated the network during 30,000 slots, which is almost 300 frames, although for the sake of simplicity, Figure 5a,b only show results for the first 2500 slots. We have seen that the behavior of the whole simulation can be summarized by analyzing this initial period.

In one side, Figure 5a shows the end-to-end delay of message $m_{ga}$ when it is alone, i.e., the only message across the network is $m_{ga}$. As it can be seen, the average time needed for the message delivery is 28 slots. On the other side, Figure 5b shows the end-to-end delay of message $m_{db}$ when it is alone. Again, results are as expected, since the average time of 18 slots is coherent with the theoretical computations. At this point, it is important to clarify the trends that both figures present in their results. In fact, we can observe that there are a constant trend, and an oscillating trend. Specifically, there is a minimum constant delay of 23 slots for the path *g*—*f*—*e*—*a* of message $m_{ga}$, and a minimum constant delay of 12 slots for the path *d*—*c*—*b* of message $m_{db}$. Moreover, there is an oscillation of 11 slots due to the difference between the frame size (11 slots) and the message, which is every 100 slots for message $m_{ga}$, and every 80 slots for message $m_{db}$. In conclusion, we can ensure that simulator behaves as expected.

### 7.3. End-To-End Delay Analysis

In this simulation we also considered the example of Section 5, the topology and delays of Figure 3, and the frame and slot allocations of Table 3. We simulated the network during 30,000 slots, and we assumed a random initial-time (in slots) to start the message transmission. Finally, the set of messages $m_{ga}$ and $m_{ag}$ are sent every 100 slots, and the set of messages $m_{db}$, $m_{bd}$, $m_{ec}$, and $m_{ce}$ are sent every 80 slots.

**Figure 6:**
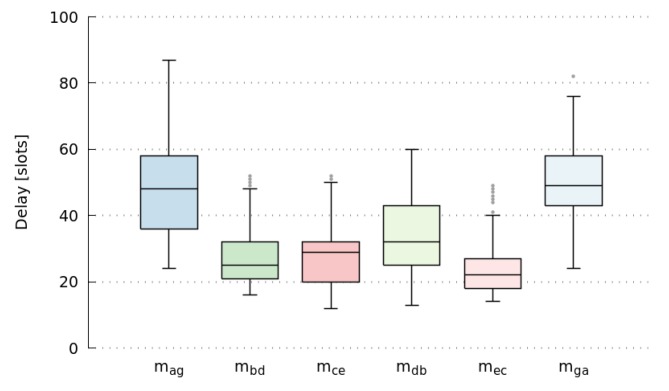
End-to-end message delays.

Figure 6 shows the end-to-end delay of messages $m_{ga}$, $m_{ag}$, $m_{db}$, $m_{bd}$, $m_{ec}$, and $m_{ce}$. It summarizes the results of ten simulations with various initial random-values. It is important to note that packet delivery rate of all simulations is 1.0, meaning that all messages have been delivered. Moreover, all of them satisfy their deadlines, as network goodput ratio is 1.0. In this case, we defined the deadline as in Equation (15).

**Figure 7:**
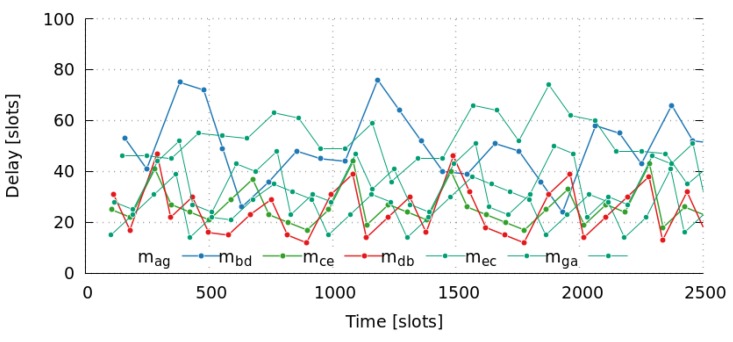
Sample of end-to-end message delays over time.

**Figure 8:**
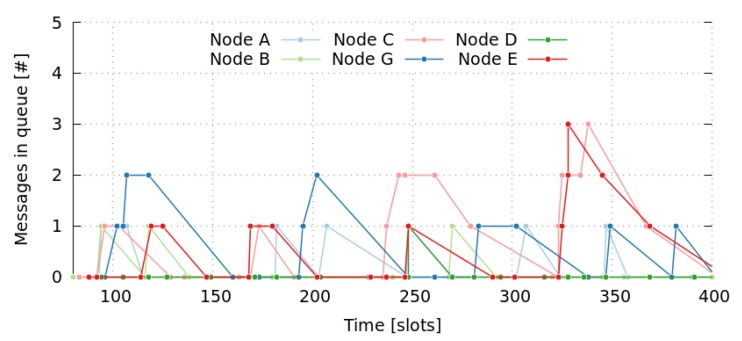
Sample of node queue length.

We can also observe that there is a high dispersion of end-to-end delay values, particularly large for messages with the highest number of hops. Moreover, it does not seem to follow any trend, as it presents continuous changes over time. In this way, Figure 7 shows the oscillating behavior of the message delays for the first 2500 slots. This can be explained by the huge number of messages that are pending to be sent by a node (own messages or in-transit messages), which is known as the Bufferbloat effect [66]. Figure 8 shows the queue occupancy (number of messages) of nodes *a* to *e* for the first 400 slots. Notice that a node can only transmit a single message per frame (11 slots). Therefore, these pending messages in queues cause the self-adaptation of messages to go through other paths, involving nodes with fewer pending messages. Table 8 shows the path usage of a message going from the same initial node to the same final node. Finally, we also observed that the delay of opposite paths do not necessarily need to be the same, as we can see in Figure 6 considering the delays of messages $m_{ga}$ and $m_{ag}$. Summarizing, we can conclude that results obtained by simulations, present even better end-to-end delay values than those that were analytically computed in Section 5.

### 7.4. Fault-Tolerance Case Study

**Figure 9:**
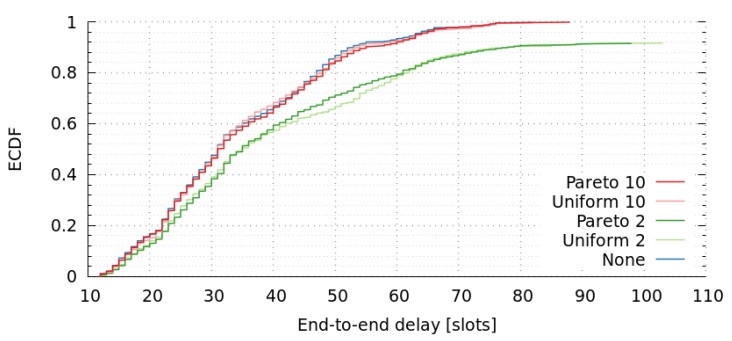
End-to-end message delays.

The same scenario of previous sections has been assumed to analyze the fault tolerance of messages in the presence of link failures (message dropping). First, we analyzed the empirical cumulative distribution function of message end-to-end delays (see Figure 9). In this figure, we assumed three different link-failure models: (1) None; (2) Uniform; and (3) Pareto. The uniform model deals with a uniform distribution that has constant probability. Unfortunately, this failure model is memoryless, which means that it does not reflect the bursty nature of failures, also known as the long-range dependency. For a more realistic model, a self-similar process such as the Pareto distribution can be used as a long-tail failure-model.

We have simulated two different approaches in terms of message dropping: (1) a fail every ten messages; and (2) a fail every two messages. This parameter is denoted as Mean Time Between Failures (MTBF). Notice that a two-message MTBF can be considered as an extreme failure-rate.

Table 9 shows that packet delivery ratio (PDR) is 1.0 or nearly 1.0 when there is a fail every 10 messages, but it decreases significantly (close to 0.92) when there is a fail every 2 messages (extreme failure-rate). Anyway, all delivered messages are within the assumed deadline. We can also observe that these failures produce a slightly increase in the end-to-end delay when it is assumed a fail every 10 messages, and a higher increase (although not important) when it is assumed a fail every 2 messages. This difference is due to the fact that, in the presence of higher failure-rates, messages deal with more paths and more unusual ones. For instance, Table 10 shows the path usage of all messages analyzed for the Pareto distribution model, with fails every 10 messages and every 2 messages. There we can observe that the most popular path has similar percentage of use in both cases, but Pareto model (that fails every 2 messages) introduces more paths with small percentage of use, compared to the other failure approach.

As explained in Section 6, we assumed an opportunistic routing-algorithm based on the epidemic model, as UW-ASN have a small traffic-intensity. If we consider any other routing model, most of the nodes that are in the middle of several paths may be overloaded. In order to demonstrate this, we have compared tree multi-hop routing-algorithms: Epidemic, Shortest-Path-First (SPF), and UnderWater Opportunistic Routing based [42] (UWOR-based).

- Epidemic: This is the proposed flooding routing scheme. Aiming to deal with message loss (due to high noise and interference of underwater scenarios) and link degradation (due to water currents and the sensor-nodes movements, the topology changes frequently).
- UWOR-based [42]: This is a simplified version of the proposal called “Unicast without ReTx”. It uses the proposed metric EEL|success(.) to find a single forwarder by taking into account both one-hop reliability and end-to-end latency, but in these simulations the T-Lohi MAC protocol has been changed by TDMA, and also some minor changes to compute the EEL|success(.) metric had been done.
- Shortest-Path-First (SPF): This is a classical routing schema that we assume as a baseline.

Table 11 shows Packet-Delivery Ratio (PDR) and network-goodput ratio of both routing algorithms, considering no link-failures and the Pareto 2 approach. There we can observe that, with no link-failures, all messages were delivered (PDR = 1.0) and their deadlines were satisfied (PDR matches network goodput ratio). Unfortunately, this is not a realistic scenario in UW-ASN.

On the other hand, when we compare Epidemic, UWOR-based, and SPF routing considering a link-failure model (Pareto 2), we can observe that PDR is below 1. Notice that this is quite good for the Epidemic-routing approach (PDR = 0.94), but significantly poor for the SPF schema (PDR = 0.72), and UWOR-based schema (PDR = 0.87). Notice also that, in both cases, the deadline of messages is satisfied (network goodput ratio matches PDR).

**Figure 10:**
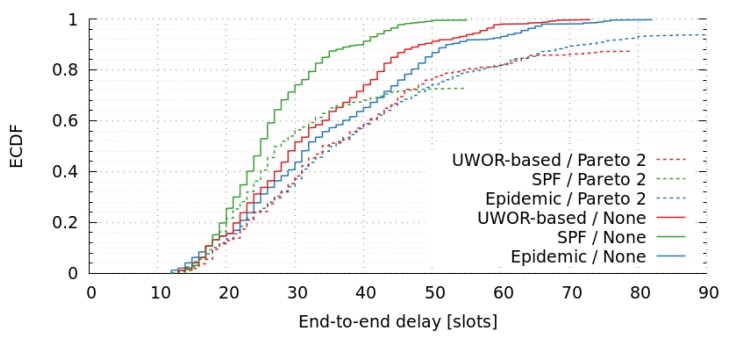
End-to-end message delays.

Figure 10 shows empirical cumulative distribution-function of end-to-end message delays for these routing strategies, with and without link-failures. The results indicate that SPF has better end-to-end delays compared to the Epidemic and UWOR-based strategies. In this case, the large number of Epidemic messages fills the queues of the nodes, thus delaying the message transmission. However, its PDR is significantly better. Therefore, this is reduced to a trade-off decision between end-to-end delay and packet-delivery ratio.

The harshness of the submarine environment makes the UW-ASN highly challenging, and underwater communications are susceptible of losing messages. Hence, our goal is that messages reach destination nodes, therefore, we choose the Epidemic approach since it prioritizes PDR over delay (within RT deadlines). Any other routing algorithm that transmits an unique message and uses a unique path, put in risk the communication given the rate of message lost in such a communication medium is usually high. In this case, the use of epidemic routing improves the transmission capability without increasing considerably the message distribution cost (in terms of energy); this is because the model uses TDMA, and therefore only the assigned slots are used in the transmission.

### 7.5. Impact of Frame Length

**Figure 11:**
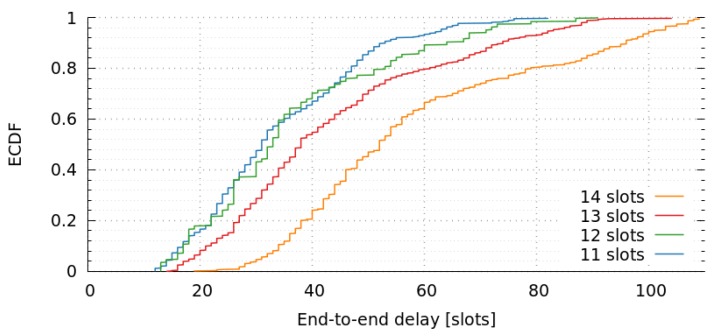
End-to-end message delays for various frame-lengths.

We maintain the same scenario as in previous sections, to analyze the impact of end-to-end delays for various frame lengths. The only variation between simulations is the frame length, which is in the range of 11 to 14 slots. Figure 11 shows the empirical cumulative distribution-function of end-to-end delays of message for various frame lengths. The results indicate that there is an important end-to-end delay increase when frame length increases. We have also observed that 14 slots is the maximum admissible value, as greater values produces poor goodput ratios. For instance, the goodput ratio of a frame length of 15 is just 0.37. In Section 6 we computed the optimum frame-length with the genetic algorithm, and we concluded that 11 was the minimum admissible value. In this section, we have simulated our approach with the minimum and higher lengths, and realized that slots of 11 or 12 achieve similar results. However, there is a significant decrease in terms of performance when we consider slots greater than or equal to 13.

## 8. Conclusions and Future Work

The use of UW-ASN is still in a developing state, far from reaching standard consensus on basic aspects like carrier frequency or modulation techniques. However, their relevance in making the development of underwater ubiquitous systems possible, guarantees the research on this topic for the years to come. The main limitation for the development of these networks has been the lack of models to represent the network performance in several application scenarios, particularly in those requiring real-time communication.

In this paper, we advance the state-of-the-art in this topic, by proposing a network analytic model that represents the behavior of UW-ASN that cover wide areas involving a few nodes (i.e., low-density networks) and using an any-to-any communication scheme. The model considers real-time message-delivery, which is an aspect little explored by the research community, and mandatory for implementing some underwater ubiquitous-solutions, such as tsunami-warning systems.

The real-time analysis for an underwater acoustic sensor network was done using two approaches. In the first one, the network is analyzed with integer linear programming techniques. The shortest path is used as routing policy combined with a message or node slot-allocation procedure in a TDMA frame. Based on this, we presented the schedulability condition for the case in which the messages are transmitted following a FIFO policy. This scheduling strategy is quite simple and requires little processing within the underwater nodes, thus reducing the computing complexity and the demand on the processors. However, better results may be obtained if some real-time priority policies are implemented, such as fixed priorities or earliest-deadline first. The performance of the network by using priority policies is left for future work.

The second approach is based on a heuristic, where messages are scheduled following a per-link processing, finding the route with shortest delay. The solution obtained improves the two-step approach of finding the SPF in the first place, and then allocating the slots within the frame. As this heuristic only considers the messages actually being transmitted, unnecessary restrictions are avoided.

In order to illustrate the applicability of the proposal, we modeled an UW-ASN for a simulated scenario where real-time transmission is necessary. Particularly, we implemented a network similar to the one required to support tsunami-warning systems, and we provided a routing proposal that helps meet the real-time requirements. This network capability increases the opportunities of developing underwater ubiquitous systems for other application scenarios such as environmental monitoring, study of marine species and climate change, biodiversity protection and defense.

Given that the network model is analytic, the designers of these systems can use software tools to quickly create, evaluate and adjust their models until reaching suitable results. Therefore, they can proceed to implement these solutions by knowing the expected performance of these systems in the real world. This clearly contributes to reducing development efforts and risks, and making the solutions more effective. An alternative to the use of the proposed model is to perform iterative design based on simulations; however, such a process even needs an UW-ASN model, and it is much more time-consuming. The next step in this initiative is to use the proposed model to design a real-world UW-ASN that helps us empirically verify the capabilities of the proposal.

## Figures and Tables

**Table 1:** Model notation.

Notation	Description
*V*	the set of nodes
*u*, *v*, *i*, *j*, *k*	nodes
N(i)	the set of neighbor nodes of *i*
Θ(u,v)	the set of edges between *u* and *v*
$t_{i}$	the slot in which node *i* transmits
$m_{ij}$	a message from node *i* to node *j*
$\tau_{uvh}$	the transmission delay between *u* and *v*
$P_{ij}$	transmission period of message from node *i* to *j*
$D_{ij}$	deadline of message from node *i* to *j*

**Table 2:** Node/time slot allocation for transmission of messages.

Node	Time Slots					
	1	2	3	4	5	6
*a*	Tx(a)				Rx(b)	Rx(d)
*b*	Tx(b)			Rx(d)	Rx(a)	Rx(c)
*c*			Tx(c)	Rx(b)	Rx(e)	
*d*	Tx(d)			Rx(b)	Rx(e)	Rx(a)
*e*		Tx(e)		Rx(d)		Rx(c)

**Table 3:** Node/Slot allocation for transmission/reception of messages.

Node	Slots										
	1	2	3	4	5	6	7	8	9	10	11
*a*				Tx(a)	Rx(b)	Rx(e)	Rx(b)	Rx(e)			
*b*	Tx(b)			Rx(e)	Rx(e)	Rx(c)	Rx(c)	Rx(a)		Rx(a)	
*c*			Tx(c)	Rx(b)	Rx(b)	Rx(f)	Rx(f)				
*d*					Tx(d)	Rx(c)	Rx(c)	Rx(g)	Rx(g)		
*e*	Tx(e)			Rx(b)	Rx(b)	Rx(f)	Rx(f)		Rx(a)		Rx(a)
*f*			Tx(f)	Rx(e)	Rx(e)	Rx(c)	Rx(c)	Rx(g)	Rx(g)		
*g*					Tx(g)	Rx(f)	Rx(f)	Rx(d)	Rx(d)		

**Table 4:** Worst case messages to be scheduled per node.

Node	Messages
*a*	$\left\{m_{ag},m_{bd},m_{ce},m_{db},m_{ec}\right\}$
*b*	$\left\{m_{ag},m_{bd},m_{ce},m_{ec},m_{ga}\right\}$
*c*	$\left\{m_{ag},m_{bd},m_{ce},m_{db},m_{ga}\right\}$
*d*	$\left\{m_{ag},m_{ce},m_{db},m_{ec},m_{ga}\right\}$
*e*	$\left\{m_{ag},m_{bd},m_{db},m_{ec},m_{ga}\right\}$
*f*	$\left\{m_{ag},m_{bd},m_{ce},m_{db},m_{ec},m_{ga}\right\}$
*g*	$\left\{m_{bd},m_{ce},m_{db},m_{ec},m_{ga}\right\}$

**Table 5:** Worst case response time per node.

Message	Nodes						
	*a*	*b*	*c*	*d*	*e*	*f*	*g*
$m_{ag}$	55	55	55	55	55	66	
$m_{bd}$	44	44	44		44	44	44
$m_{ce}$	44	44	44	44		44	44
$m_{db}$	44		44	44	44	44	44
$m_{ec}$	44	44		44	44	44	44
$m_{ga}$		55	55	55	55	66	55

**Table 6:** Worst case end-to-end delay.

Path	Delay
aefg	210
abcdg	275
abefg	275
abcfg	286
abefcdg	407

**Table 7:** Genetic Algorithm and Exact Solution.

Instance	ILP		GA		GA Reduced	
	L	CT	L	CT	L	CT
I	19	400	19	0.43	14	0.63
II	–	∞	12	93	10	2.90
III	9	3100	9	1.06	9	1.40
IV	11	100	11	0.4	8	0.50
V	13	38,880	13	0.43	10	1.00
VI	14	2000	14	0.58	10	0.70
VII	9	12,000	9	2.47	9	1.08
VIII	12	5000	12	0.39	9	0.51

**Table 8:** Path usage.

Message	Path	Usage (%)
$m_{ag}$	abcg	18%
$m_{ag}$	aefg	82%
$m_{db}$	dcb	100%
$m_{ce}$	cbe	94%
$m_{ce}$	cfb	6%
$m_{bd}$	bcd	100%
$m_{ec}$	ebc	94%
$m_{ec}$	efc	6%
$m_{ga}$	gdca	20%
$m_{ga}$	gfca	11%
$m_{ga}$	gfea	69%

**Table 9:** Path usage.

Metric	None	Uniform 10	Uniform 2	Pareto 10	Pareto 2
packet delivery ratio (PDR)	1.0	0.998	0.918	1.0	0.916
network goodput ratio	1.0	0.998	0.918	1.0	0.916

**Table 10:**
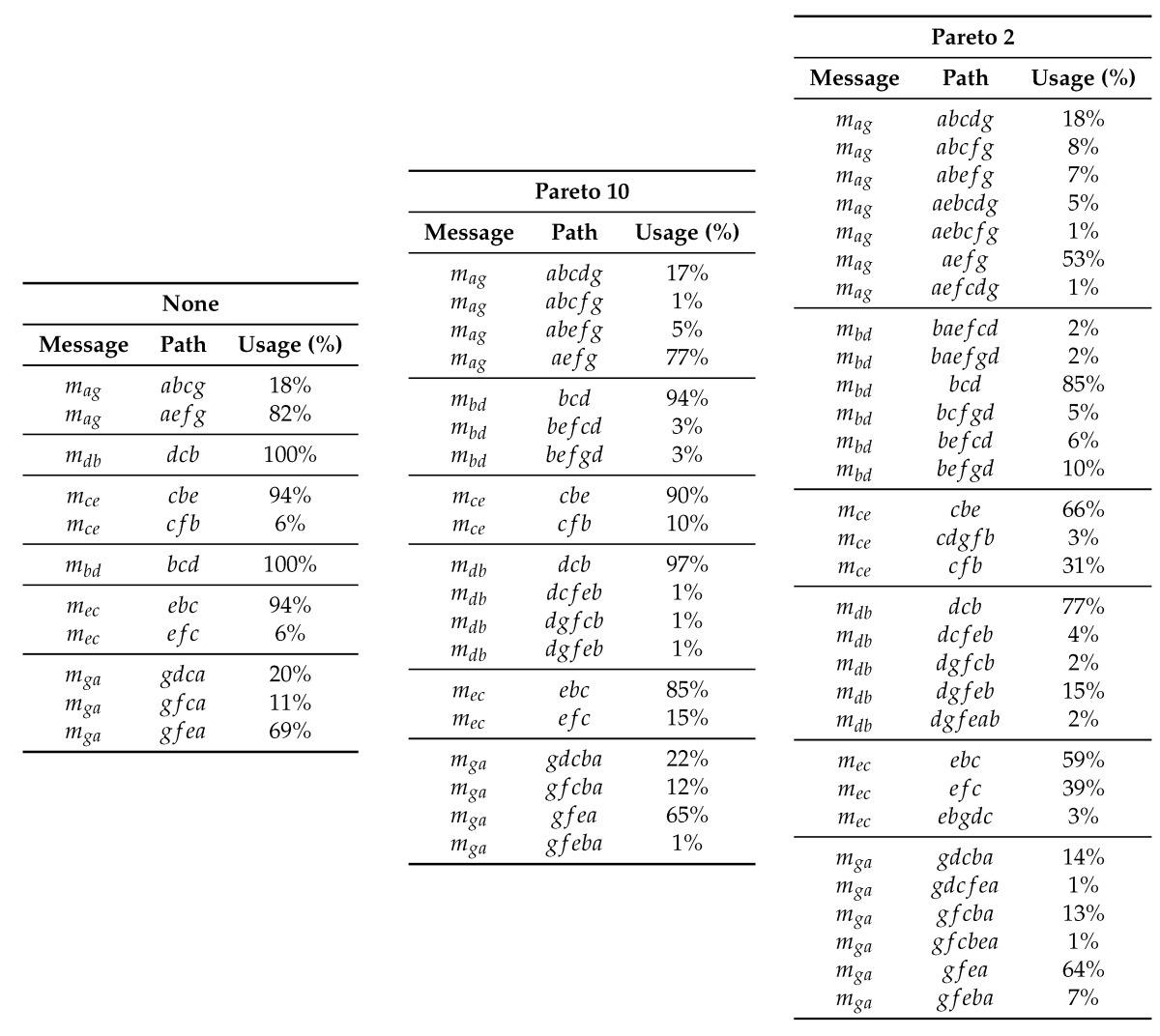
Path usage.

**Table 11:** Epidemic, UWOR-based, and Shortest-Path-First (SPF) routing comparison.

Metric	Epidemic / None	UWOR-Based / None	SPF / None
packet delivery ratio (PDR)	1.0	1.0	1.0
network goodput ratio	1.0	1.0	1.0
**Metric**	**Epidemic / Pareto 2**	**UWOR-Based / Pareto 2**	**SPF / Pareto 2**
packet delivery ratio (PDR)	0.94	0.87	0.72
network goodput ratio	0.94	0.87	0.72
